# The More, the Better?! Multiple vs. Single Jobholders’ Job Satisfaction as a Matter of Lacked Information

**DOI:** 10.3389/fpsyg.2017.01274

**Published:** 2017-07-26

**Authors:** Maria U. Kottwitz, Lena Hünefeld, Benjamin P. Frank, Kathleen Otto

**Affiliations:** ^1^Faculty of Psychology, Work and Organizational Psychology, Philipps University of Marburg Marburg, Germany; ^2^German Federal Institute for Occupational Safety and Health Dortmund, Germany

**Keywords:** multiple jobbers, information, job satisfaction, atypical employment, work design

## Abstract

In recent decades, the working world has changed dramatically and rising demands on flexibility make the coordination of personal and professional life more difficult. Therefore, it is important that the incumbents are in possession of all necessary information concerning their job. This might be a key issue to remain satisfied. Simultaneously, atypical forms of employment have substantially increased in the labor market; one such form is holding more than one job. While the motives might differ from needing an additional income to broadening job opportunities, practicing several jobs requires coordination and thus, being informed. Building on research regarding organizational constraints and role ambiguity, we hypothesize that the paucity of information is negatively related to (dimensions of) job satisfaction. This effect should be stronger for multiple as compared to single jobbers; specifically when considering the job satisfaction with the social climate, given that being informed by others is an important factor in the coordination of several jobs. Data taken from the BiBB/BAuA-Employment-Survey provide a sample of 17,782 German employees (54% women), including 1,084 multiple jobbers (59% women). Job satisfaction was measured as employees global satisfaction and their satisfaction with facets dimensions: the social climate, structural working conditions, personal growth opportunities, and material incentives they receive for their work. Paucity of information was measured by the frequency of lacked information. Our study indicated that paucity of information was negatively related to both, global and all facets dimensions of job satisfaction. Multiple regression analyses further revealed interaction effects of paucity of information and form of employment. Specifically, the negative correlation of paucity of information with global as well as satisfaction with the social climate was stronger for employees’ holding more than one job. These results were independent of age, gender, organizational tenure, working hours, socioeconomic occupational status, as well as important working conditions (workload and autonomy). Incumbents with less paucity of necessary job-related information are more satisfied, especially when they hold multiple jobs. Supervisors and colleagues are advised to provide all necessary information and to ensure that employees retain it.

## Introduction

Nowadays, intensification of work and raising demands are common consequences for the working population (Kompier, 2006). New technologies, especially new communication technologies, have led to an increasing complexity and diversity of tasks in recent years (Neubach and Schmidt, 2006). Therefore, employees must be able to manage and coordinate a great deal of information. Communication (containing the aspect of providing information) is known to be an important factor shaping persons’ health, attitudes and behavior (Pincus, 1986; Langan-Fox, 2002). Moreover, atypical employment forms are a growing global phenomenon (Keller and Seifert, 2013). It is associated with flexibility requirements – in particular, when employees hold more than one job. Do multiple jobbers differ from single jobholders in the way that information might be even more important for them as compared to single jobbers?

Overall, balancing out different personal and professional life domains became an important topic (Lunau et al., 2014; Nohe et al., 2015). To prevent conflicts between life domains or contexts, especially, strategies that help employees to coordinate their responsibilities, activities, and obligations are needed (Voydanoff, 2005; Van Dyne et al., 2007). Organizations generate much information that is not generally accessible to every employee although some of these pieces of information are necessary to fulfill the task (Langan-Fox, 2002). As an important aspect of organizational support, providing information is linked to commitment and performance (e.g., Bishop et al., 2000; Cortini, 2016; Cortini et al., 2016). Accurate information about one’s performance and behavior facilitates coordination by effectively setting behavior altering goals for oneself (Manz, 1986; Manz and Neck, 2004). In contrast, a lack of necessary information aggravates coordination. Adverse work conditions such as lack of information are called job stressors as they increase the likelihood of employees to experience stress (Beehr and Franz, 2008). Consequently, we undertook this research to study employee reactions when they perceive a paucity of information; that is when they have incomplete, obsolete, or poorly timed information.

Research on the lack of information as a stressor has mainly focused on medical teams (Hunziker et al., 2010). For example, medical teams tend to discuss shared information but not unshared information (Larson et al., 1996). Unfortunately, necessary information is not always available (on time). However, efficient communication is an important factor among team members (Kurmann et al., 2012) that goes beyond medical teams. As an aspect of performance- or organizational constraints, the paucity of information prevents employees from translating ability and effort into job performance (Peters and O’Connor, 1980; Spector and Jex, 1998). Imagine a scenario in which John, a secretary, needs information from a manager to complete a form, but the manager is unavailable. Although, John has the skills, abilities and capacities to carry out this task, the paucity of information makes the accomplishment more difficult, if not impossible. In addition, paucity of information is included in the concept of role ambiguity by dealing with unclear expectations (Katz and Kahn, 1978); for instance, due to a lack of feedback. Thus, paucity of information could not only be seen as an obstacle of task regulation, but also as regulation uncertainty referring to uncertainty about how to reach the goal (Sonnentag and Frese, 2012). Organizational constraints (Pindek and Spector, 2016) and role ambiguity (Örtqvist and Wincent, 2006; Fried et al., 2008; Eatough et al., 2011) are both known to be related to impaired job satisfaction. We therefore hypothesize that paucity of information will be negatively related to job satisfaction (Hypothesis 1).

Specifically, receiving too little information to perform well should affect one’s satisfaction with the information system (i.e., colleagues or supervisors) as well as with structural conditions hindering transmission of information (Churchill et al., 1976). Moreover, as the paucity of information hinders goal attainment, it should affect the satisfaction with personal gains, in sense of personal growth opportunities (e.g., as skill utilization is supposed to be blocked) as well as material incentives one could have had reached (Peters and O’Connor, 1980). Thus, the paucity of information will be negatively related to:

(H1a) Satisfaction with the social climate at work referring to being included and connected to colleagues and supervisor (satisfaction with the social climate),
(H2a) Satisfaction with structural working conditions (i.e., the working materials, physical conditions, working hours) related to the context of the task that has to be fulfilled (satisfaction with structural conditions),
(H3a) Satisfaction with developmental experiences that facilitate the growth in needed skills (satisfaction with growth opportunities), and
(H4a) Satisfaction with the material incentives (i.e., with respect to career opportunities and income) a person receives at work (satisfaction with material incentives).

Research on multiple jobbers is limited (Zickar et al., 2004; Marucci-Wellman et al., 2016) although every twentieth German employee holds more than one paid job (German Federal Statistical Office, 2015). Holding multiple jobs is known to affect work-life balance (McClintock et al., 2004; Sliter and Boyd, 2014). In general, long working hours or working overtime are at the cost of recovery (Geurts, 2014). Compared with single jobbers, multiple jobbers spend more time working (McClintock et al., 2004; Sliter and Boyd, 2014), endure longer working hours or overtime work. Beyond the quantitative workload, multiple jobbers must expend additional effort in coordinating and balancing different personal and professional life domains (McClintock et al., 2004; Olos and Hoff, 2007). Recall the scenario of John, the secretary, struggling with a lack of information. Perhaps the solution is to wait until the manager is available, or to ask others who might have answers, or to search for documents including the missing information. Whatever solution is selected, time is invested. Such solutions are particularly difficult when coordinating more than one job. Working overtime in one’s first job to accomplish a task might be impossible without causing negative consequences for the second job. Based on current studies we assume that multiple job holding and paucity of information intensify one another’s adverse effects (De Cuyper and De Witte, 2005; Virtanen et al., 2011). Thus, we expect a stronger association between paucity of information and job satisfaction for multiple as compared to single jobbers (Hypothesis 2).

Information transmission is done (either directly or indirectly) by communication (Langan-Fox, 2002). Recent research on instrumental support show that supervisor support helps to coordinate between different life domains (Tucker et al., 2016). Task assistance (i.e., to help the person to get the task done) as a part of instrumental support has been most strongly associated with job satisfaction (Colbert et al., 2016). Giving information could even be seen as some kind of immaterial reward, if it is shared exclusively during small groups (Van Yperen, 1998). Moreover, social support – such as giving information to manage a task – provides an inherent social message about how the person is seen by (significant) others (Cobb, 1976; Semmer et al., 2007). Instrumental social support (i.e., by assisting with problem solving through giving information) is often valued for the inherent expression of appreciation (e.g., Semmer et al., 2008). In contrast, the paucity of necessary information might be seen as a sign of disrespect threatening the self (cf. stress as offense to self; Semmer et al., 2007). If the information is essential to complete tasks, employees will perceive that the transmitter, whether supervisor or colleagues, is responsible for providing the information (Tschan et al., 2009). Tschan et al. (2009) even point to the risk of illusory transactive memory, i.e., that group members expect experts who hold important information to be aware to inform them, if necessary leading to the risk that group members do not seek for additional information themselves. In general, providing support is known to increase a person’s perceived control over his or her environment, helping the person to coordinate different obligations (Greenberger and Strasser, 1986; Thompson and Prottas, 2006). Especially in case of uncertain conditions, employees rely on supervisors and colleagues with respect to information seeking, such as in case of organizational change (Van der Voet et al., 2014; Tanner and Otto, 2016) or being a newcomer in the organization (Wolfe Morrison, 1993). Similarly, spending additional effort in information seeking activities is particularly difficult when coordinating more than one job. While there might be no difference in the attribution to structural conditions, growth opportunities or material incentives, if necessary information is not available (on time) to accomplish the task, especially multiple jobbers might attribute this lack of information to colleagues and supervisors who should inform them. Considering that holding multiple jobs requires additional coordination efforts, multiple jobbers should show a more negative association between the paucity of information and satisfaction with the social climate (H2a).

## Materials and Methods

### Participants and Procedure

Data for the current analyses refer to the BiBB/BAuA Employment Survey of the Working Population on Qualification and Working Conditions in Germany in 2012. This representative survey was conducted by the Federal Institute for Vocational Education (BIBB) and the German Federal Institute for Occupational Safety and Health (BAuA). Altogether 20,036 volunteers above 15 years of age who worked at least 10 h per week completed a highly standardized interview by trained interviewers on the phone (Rohrbach-Schmidt and Hall, 2013).

Interviewers asked participants whether they had one or more gainful occupations to differentiate single and multiple jobbers. Afterward, participants were asked to report which occupational activity they currently pursued to be denoted as main activity according to the time they spend in this activity. All following questions about working conditions and context were focused on this main activity. As dependent employees and business founders might differ in organizational information processes, we focused on main activities within a dependent employment relationship. Data of 35 participants were excluded due to missing information with respect to employment status. To avoid potentially confounding effects of the employment status, we excluded 71 family workers and 2,129 self-employed persons (including free-lance workers and independent contractors). Moreover, we excluded 19 student research assistants/ student employees. The final sample thus consisted of 17,782 participants (53.87% women), including 3,142 blue collar workers, 13,075 white collar workers, 65 persons who were unable to decide between those two categories, and 1,500 civil servants. Among the total participants, 1,084 (6.1%) were multiple jobbers (59.23% women). Participants overall had a mean age of 45.61 years (*SD* = 10.57): single jobbers had a mean age of 45.71 years (*SD* = 10.56); multiple jobbers had a mean age of 43.97 (10.56).

### Measures

#### Job Satisfaction

Job satisfaction was measured both by single-item measure of global job satisfaction and by employees’ satisfaction with various facets of his/her job, as they are argued to be conceptually different (Scarpello and Campbell, 1983; Faragher et al., 2005). Participants were asked how satisfied they are regarding various aspects of their main occupational activity and, afterward, about their global job satisfaction (“And now, as an overall summary: How satisfied are you with your entire occupational activity?”). Single-item measures of global job satisfaction are as reliable and valid as measures containing different facets (Wanous et al., 1997).

Each facet was measured by a single item. Confirmatory factor analysis was used to classify facets into higher-order dimensions (see below): (1) satisfaction with rather structural aspects of the task (working hours, physical working conditions and work equipment, including furniture and software; three items), (2) satisfaction with manifest aspects referring to material incentives (income and career opportunities; two items; *r* = 0.36, *p* < 0.001) as well as satisfaction with psycho-social functions – (3) satisfaction with the social climate (including the working climate and the direct supervisor; two items; *r* = 0.55, *p* < 0.001) and (4) satisfaction with developmental experiences that facilitate growth (the opportunities to apply skills, the opportunities for continuing training and learning and the type and content of work; three items). The response scale ranged from 1 (not satisfied) to 4 (very satisfied). Cronbach’s alpha was 0.55 with respect to satisfaction with structural working conditions and 0.71 for satisfaction with personal growth.

To validate the measure, we compared the assumed four-factor solution with a global one-factor job satisfaction model in which all items were modeled to load on the global factor. The confirmatory factor analysis including the four factors described above resulted in an acceptable fit for the multi-factor solution. The chi-squared goodness-of-fit index (χ^2^) failed to indicate a good fit between the observed covariance matrix and the hypothesized model [χ^2^(29,15,410) = 23,88.83, *p* < 0.001], probably because of the large sample size. The validity of a model must be assessed by more than one index (Tanaka, 1993). The root mean square error of approximation (RMSEA) refers to the discrepancy per degree of freedom for the model. The obtained value was 0.07, an appropriate fit as its value was lower than 0.08 (Browne and Cudeck, 1993). The adjusted goodness-of-fit index (AGFI) explains the overall variation of the proposed model. The value was 0.94 and thus exceeded 0.90, constituting a good fit (Jöreskog and Sörbom, 1993). In addition, the standardized root mean square residual (SRMR; i.e., a measure of the average of the fitted residuals) was 0.04, presenting an excellent fit (Jöreskog and Sörbom, 1993). In summary, the indices indicated a good fit between the data and our constructs, validating our measures. The second model was composed of a general factor. The confirmatory factor analysis failed to support the validity of the measures; the fit indices were χ^2^(35,15410) = 6906,67, *p* < 0.001; RMSEA was 0.11; AGFI was 0.87. Only the value of SRMR was 0.06 representing an acceptable fit. Consequently, the first model composed of various factors was a more appropriate fit than the global one-factor model.

#### Paucity of Information

Paucity of information was measured by a single item: “In your workplace, how often do you lack the information you need to perform your work correctly?,” The response scale ranged from 1 (never) to 4 (often).

#### Control Variables

The current study aims to demonstrate the importance of a paucity of information controlling for a set of context and person variables as well as working conditions related to job satisfaction. Besides gender and age of a person, the organizational context might affect a person’s job satisfaction. We therefore controlled for organizational tenure, working hours, and the International Socio-Economic Index of occupational status (ISEI; Ganzeboom et al., 1992) of the main activity. Organizational tenure reflects the work experience related to the main activity. Work experience is known to be positively related to job knowledge (Schmidt et al., 1986) and facilitates job crafting (Niessen et al., 2016). Both – age and tenure – tend to be positively related to job satisfaction (e.g., Ng and Feldman, 2010; Costanza et al., 2012). Participants were asked to report their job experience (in years) within the organization of the main activity. In addition, we controlled for working hours of the main activity as they affect the duration that one is exposed to a (stressful) working situation. Moreover, employees might differ in their job satisfaction due to their socio-economic occupational status. Existing studies show, that comparatively less-educated workers tend to have inferior working conditions, low income levels, and job insecurity (Kalleberg et al., 2000; Zytionoglu and Muteshi, 2000). The ISEI is an objective measure for socio-economic status based on the International Standard Classification of Occupations (ISCO-88). It combines weighted averages of the income and education of incumbents of each classified occupation (for detailed information see: Ganzeboom et al., 1992).

Moreover, we controlled for workload and autonomy as central working conditions (Karasek, 1979) that are known to be related to job satisfaction (Van der Doef and Maes, 1999). As task-related stressor, workload hinders the fulfillment of the task (Sonnentag and Frese, 2012). Autonomy is typically conceptualized as a key work resource helping to coordinate demanding work schedules (Rau and Hyland, 2002). Workload and autonomy were each assessed by single items (“How often does it happen in your occupational activity …”; workload: “that you have to work under strong pressure of time or performance?”; autonomy: “that you can plan and schedule your work on your own?”). The response scales ranged from 1 (never) to 4 (often).

### Statistical Analyses

We conducted hierarchical regression analyses including variables in three different blocks. In step one, control variables (including context and person variables as well as working conditions) were entered. Second, we entered the paucity of information and holding multiple jobs (main effect model). In step three, we entered the interaction term (interaction effect model). All predictor variables were centered at their grand mean to facilitate the interpretation of effects (Aiken et al., 1991). To confirm our first hypothesis (H1, H1a-d), paucity of information should have a significant main effect on job satisfaction. To confirm our interaction hypothesis (H2, H2a), the interaction term must be significant, and the pattern of the simple slopes must reveal stronger negative effects for multiple jobbers. We calculated simple slope tests using an online tool by Preacher, Curran, and Bauer (Preacher et al., 2006). Simple slopes involve the regression equation for the level of the paucity of information depending on the form of employment (multiple or single jobbers) and test whether the respective slope is different from zero.

## Results

### Descriptive Results

**Table 1** shows the descriptive statistics. As expected, the paucity of information was negatively correlated with general job satisfaction and with all dimensions of job satisfaction. Altogether, 6% of the participants reported to hold more than one job. Interestingly, holding more than one job was unrelated to paucity of information. However, employees who held multiple jobs reported less satisfaction with their jobs in general, their personal growth opportunities, and their material incentives. In addition, all control variables were significantly associated with job satisfaction in general and/or satisfaction with job dimensions. Specifically, general job satisfaction was related to all context and working conditions but unrelated to age and gender. Further exceptions comprised tenure being unrelated to satisfaction with structural conditions, workload and age with respect to satisfaction with growth opportunities, and gender being unrelated to satisfaction with the social climate.

**Table 1:** Means (M), Standard Deviations (SD), and Zero-Order Correlations of the Study Variables.

Variable	*M*	*SD*	1	2	3	4	5	6	7	8	9	10	11	12	13
(1) Global job satisfaction	3.18	0.59	-												
**Dimensions of job satisfaction**											
(2) Social climate	3.12	0.67	0.47**	-											
(3) Structural conditions	2.94	0.51	0.47**	0.40**	-										
(4) Growth opportunities	3.07	0.53	0.56**	0.42**	0.43**	-									
(5) Material incentives	2.70	0.66	0.43**	0.29**	0.38**	0.43**	-								
(6) Paucity of information	2.26	0.90	-0.24**	-0.32**	-0.24**	-0.21**	-0.17**	-							
(7) Multiple jobs^a^	0.06		-0.03**	0.00	-0.01	-0.02**	-0.06**	-0.01	-						
**Context variables**															
(8) Tenure (years)	14.21	11.17	0.03**	-0.08**	0.01	0.05**	0.12**	0.02**	-0.08**	-					
(9) Working hours (week)	38.05	11.20	-0.02*	-0.08**	-0.10**	0.09**	0.05**	0.13**	-0.08**	0.07**	-				
(10) ISEI	47.06	15.19	0.08**	0.03**	0.13**	0.15**	0.13**	0.06**	-0.02*	0.08**	0.16**	-			
**Person variables**															
(11) Gender^b^	0.46		-0.01	-0.01	0.03**	0.02*	0.05**	0.07**	-0.03**	0.05**	0.39**	-0.01	-		
(12) Age	45.61	10.57	0.01	-0.06**	-0.02*	0.01	0.03**	-0.03**	-0.04**	0.50**	-0.03**	0.01	-0.03**	-	
**Working conditions**															
(13) Workload	3.36	0.81	-0.10**	-0.14**	-0.16**	0.00	-0.06**	0.22**	-0.01	0.07**	0.25**	0.16**	0.05**	-0.01	-
(14) Autonomy	3.54	0.89	0.13**	0.09**	0.16**	0.19**	0.13**	0.02**	0.01	0.09**	0.09**	0.24**	-0.00	0.04^∗∗^	0.09^∗∗^

*Sample size: *N* ≤ 17,782 employees. ^a^0 = no, 1 = yes, ^b^0 = woman, 1 = man. ^∗^*p* ≤ 0.05; ^∗∗^*p* ≤ 0.01 (2-tailed).*

### Test of Hypotheses

**Table 2** shows results of the regression analyses. In the main effect model, we examined the effect of paucity of information on job satisfaction, controlling for personal and context variables as well as workload and job autonomy as important working conditions. Paucity of information was negatively associated with general job satisfaction and with all dimensions of job satisfaction, confirming H1 and H1a to H1d. In line with Hypothesis 2, paucity of information and multiple-job holding significantly interacted to predict global job satisfaction. The interaction effect was also significant with respect to satisfaction with the social climate (H2a).

**Table 2:** Summary of multiple regression analysis predicting global job satisfaction and job dimensions satisfaction.

		Global job satisfaction			Satisfaction with the social climate			Satisfaction with structural conditions		
Step	Variable	*B* (final)	*SE_B_*	*t*	*B* (final)	*SE_B_*	*t*	*B* (final)	*SE_B_*	*t*
1	**Context variables**									
	Tenure (years)	0.001	0.000	3.01**	-0.003	0.001	-6.68**	0.001	0.000	2.14**
	Working hours (per week)	0.000	0.000	0.80	-0.004	0.001	-7.94**	-0.006	0.000	-14.91**
	ISEI	0.003	0.000	8.52**	0.002	0.000	5.02**	0.005	0.000	18.31**
	**Person variables**									
	Gender^a^	0.004	0.010	0.42	0.034	0.011	3.09**	0.086	0.008	10.55**
	Age	-0.001	0.000	-1.70	-0.003	0.001	-5.32**	-0.002	0.000	-4.38**
	**Working conditions**									
	Workload	-0.090	0.006	-15.97**	-0.114	0.006	-17.68**	-0.107	0.005	-22.42**
	Autonomy	0.085	0.005	16.61**	0.083	0.006	14.30**	0.089	0.004	20.53**
2	**Main effect model**									
	Paucity of information	-0.154	0.005	-31.65**	-0.230	0.005	-42.28**	-0.122	0.004	-29.43**
	Multiple jobs^b^	-0.071	0.018	-3.91**	-0.035	0.020	-1.75	-0.042	0.015	-2.70**
3	**Interaction effect model**						
	Interaction	-0.044	0.019	-2.27*	-0.048	0.022	-2.24*	-0.001	0.016	-0.091
	*R*^2^ final model (Adj *R*^2^)	0.090^∗∗^ (0.089)			0.133^∗∗^ (0.133)			0.129^∗∗^ (0.129)		

		**Satisfaction with growth opportunities**			**Satisfaction with material incentives**		
**Step**	**Variable**	***B* (final)**	***SE_B_***	***t***	***B* (final)**	***SE_B_***	***t***

1	**Context variables**						
	Tenure (years)	0.002	0.000	3.41**	0.008	0.001	15.27**
	Working hours (per week)	0.003	0.000	8.41**	0.001	0.000	2.91**
	ISEI	0.004	0.000	13.65**	0.005	0.000	14.03**
	**Person variables**						
	Gender^a^	-0.012	0.009	-1.42	0.046	0.011	4.28**
	Age	-0.001	0.000	-2.10**	-0.003	0.001	-5.53**
	**Working conditions**						
	Workload	-0.032	0.005	-6.24**	-0.082	0.006	-13.14**
	Autonomy	0.095	0.005	20.72**	0.074	0.006	13.09**
2	**Main effect model**						
	Paucity of information	-0.137	0.004	-31.15**	-0.132	0.005	-24.13**
	Multiple jobs^b^	-0.034	0.016	-2.09*	-0.134	0.020	-6.62**
3	**Interaction effect model**						
	Interaction	-0.021	0.017	-1.22	-0.022	0.022	-1.03
	*R*^2^ final model (Adj *R*^2^)	0.104^∗∗^ (0.103)			0.084^∗∗^ (0.084)		

*Sample size: *N* ≤ 17,782 employees. B, unstandardized regression coefficient, SE_B_, standard error, *t* = *t*-value. All predictors were centered at their mean. ^a^0 = woman, 1 = man, ^b^0 = no, 1 = yes. ^∗^*p* ≤ 0.05; ^∗∗^*p* ≤ 0.01 (2-tailed).*

**FIGURE 1:**
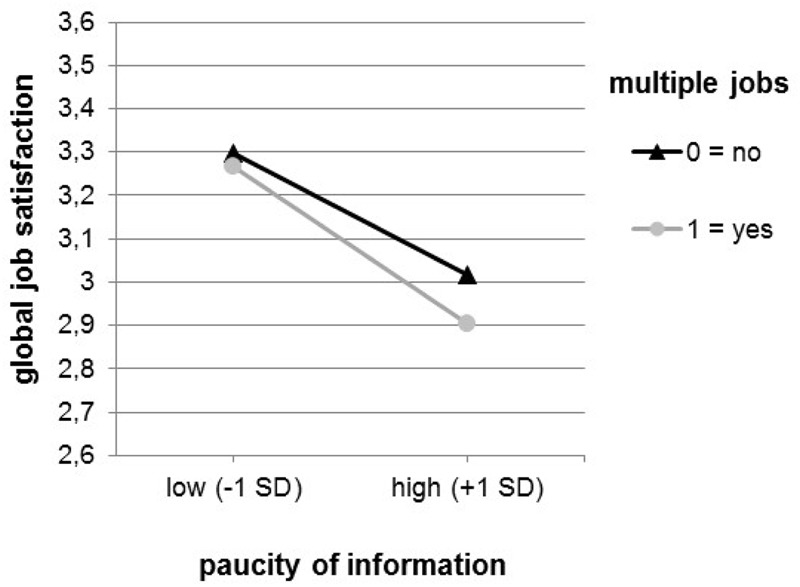
Interaction between paucity of information and form of employment predicting global job satisfaction.

**FIGURE 2:**
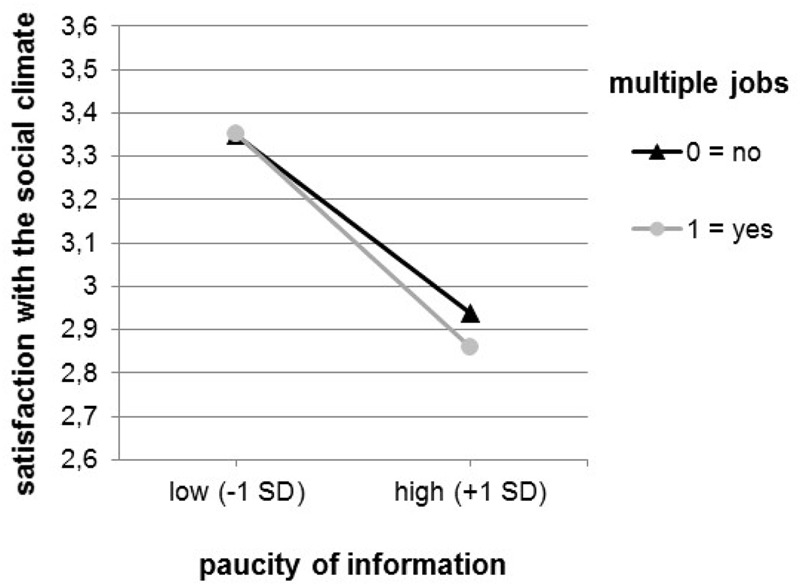
Interaction between paucity of information and form of employment predicting job satisfaction with the social climate.

According to simple slope tests, within the scope of global job satisfaction, the association between the paucity of information and satisfaction was stronger for multiple (*b* = -0.201, *p* < 0.01) as compared to single jobbers (*b* = -0.157, *p* < 0.01). A similar pattern was found with respect to job satisfaction with the social climate (multiple jobbers: *b* = -0.275, *p* < 0.01; single jobbers: *b* = -0.227, *p* < 0.01). This pattern aligns with Hypothesis 2 and Hypothesis 2a (**Figures 1, 2**). In line with statistical tradition (Heinrichs et al., 2003), we chose values of 1 SD below and above the sample mean.

### Additional Analyses

To rule out the possibility that our findings were caused by the amount of time employees spent at their main occupational activity and that lower working hours and not multiple jobs shape the relation of paucity of information and job satisfaction, we examined working hours to moderate the negative association between the paucity of information and job (dimensions) satisfaction. No significant interaction effect with respect to global job satisfaction (*b* = 0.000, *p* = 0.907) and to satisfaction with the social climate (*b* = -0.001, *p* = 0.208) occurred. In addition, no significant interaction occurred with respect to satisfaction with material incentives and growth opportunities, but with respect to structural working conditions (*b* = -0.002, *p* < 0.01).

## Discussion

### Interpretation of Results

In this study, we used research on organizational constraints and role ambiguity to build our proposals that paucity of information is negatively associated with global job satisfaction and job dimensions satisfaction. These associations were assumed to be stronger for multiple compared to single jobbers; especially with respect to satisfaction with the social working climate. Based on a representative sample of 17,782 German employees, we found the paucity of information to be negatively related to job satisfaction in general as well as with respect to the dimensions of material incentives, structural conditions, growth opportunities, and satisfaction with the social climate.

One of the most frequently studied outcomes of job stressors is job satisfaction (Dormann and Zapf, 2002; Faragher et al., 2005; Pindek and Spector, 2016). It is seen as a component of psychological well-being (Diener et al., 2003) referring to the extent to which a person likes or dislikes his or her job (Spector, 1997). Beside material incentives, work or a job also allows social contacts and appreciation, gives structure, maintains and advances our skills, and is part of our identity (Jahoda, 1983; Semmer and Meier, 2014). Occupational success is bound to the fulfillment of one’s working task to the extent that this fulfillment allows the accomplishment of valued goals (c.f. Grebner et al., 2010). Paucity of information hinders employees to accomplish their tasks (organizational constraints), as well as it leads to uncertainty about how to fulfill the task (role ambiguity) which in turn impairs job satisfaction (Örtqvist and Wincent, 2006; Fried et al., 2008; Eatough et al., 2011; Pindek and Spector, 2016). Considering that the paucity of information hinders the employee to fulfill the task it also hinders the person to accomplish valued goals in sense of personal growth opportunities as well as material incentives as well as leading to frustration (Peters and O’Connor, 1980). In line with these considerations, we found the paucity of information to be negatively related to job satisfaction in general as well as to all dimensions of it. Especially in a fast changing environment, managing and coordination information seems to be an important challenge. Moreover, aspects of working conditions (such as a supportive climate or the paucity of information) or even tasks that have to be fulfilled might appear in a different light due to their inherent social message (cf., Semmer et al., 2007). Appreciation is an important element of social support (Semmer et al., 2007) referring to the perception “that one is worthwhile, capable, and a valued member of a group of individuals” (Sarason et al., 1996, p. 21). In other words, providing support (e.g., in terms of giving information) reveals that these colleagues and supervisor care about the employee. It might increase the sense of control and the employee might feel obligated to reciprocate. Thus, social support is positively related to employees’ commitment and performance (Bishop et al., 2000; Allen and Shanock, 2013; Cortini, 2016; Cortini et al., 2016). Supporting the person to fulfill the task was found to be most strongly associated with job satisfaction (Colbert et al., 2016). In contrast, behavior or assigned tasks that indicate disrespect or lack of appreciation as its social message constitutes a threat to the person by offending the self (e.g., Semmer et al., 2007). Recall again the scenario of John, searching for information that should be already there. This might be seen as an unnecessary task that would not exist, if things were organized differently. Perhaps he thought this task should be done by someone else because it does not correspond to his occupational role (i.e., an unreasonable task). Previous research found such illegitimate tasks to be related to stress-related behavioral (e.g., counterproductive work behavior; Semmer et al., 2010) and cognitive-emotional (e.g., lower job satisfaction; Stocker et al., 2010; Björk et al., 2013; Eatough et al., 2015) changes. Thus, providing adequate information might become of particular importance in the sense of goal attainment, organizational support, and legitimacy of task assignment. It constitutes a positive step toward employee commitment, performance and satisfaction. According to our results, the paucity of information seems to be practically significant over and above workload and autonomy that are known to be important working conditions related to job satisfaction (Van der Doef and Maes, 1999). It must, however, be critically stated that as long as the person succeeds in the task, the paucity of information could also be linked to a sense of pride and satisfaction: John might be proud of managing the task without proper information. It would be advisable for future research to focus on daily events of lacked information and job satisfaction taking into account if the person successfully accomplished the task.

Work is a major cause of stress for many people in a stressful world. For jobholders who must coordinate multiple jobs, to be aware of needed information seems to be especially important. In line with our expectations, we found multiple jobbers more likely to be less or not satisfied with their job in general as well as with the social climate when they perceived a lack of information. One core element of adequate information processes is the interaction with supervisors and colleagues and the embeddedness in work-related social networks. It is plausible that employees holding multiple jobs have less time to seek out information, but concentrate on the specific task (cf., Wolfe Morrison, 1993). If necessary task-related information is not given, employees might rely on their colleagues or supervisor to inform them (Tschan et al., 2009). Transmission of information represents a central management duty – especially under conditions of uncertainty. As uncertainty associated with change is often based on missing or, respectively, failed communication, communication is a main concern in the research of change management and implementation (for an overview see Grant and Marshak, 2011). Particularly in times of organizational change, supervisors play a central role in communicating visions, transmission of goals and norms to employees, and providing a mutual high quality communication (e.g., Van der Voet et al., 2014; Tanner and Otto, 2016). In addition, research on semi-autonomous teams indicates that informal communication among colleagues could compensate for inefficient information systems (Morgeson et al., 2006). As mentioned above, social support (e.g., by providing information) is known to increase a persons’ perceived control over their environment (Greenberger and Strasser, 1986; Thompson and Prottas, 2006). In contrast, employees are less satisfied with the social working climate when they lack information, especially multiple jobbers who need control to coordinate more than one job. A recent meta-analysis showed that contingent workers, another type of atypical workers, had lower job satisfaction than permanent workers (Wilkin, 2013). Even if multiple jobbers receive salaries similar to those of some colleagues holding single jobs, they react similarly to contingent workers in that they might feel less identified and integrated because of switching between different professional lives. Future research should consider social integration as a core component in studying multiple jobbers. A lack of social embeddedness – especially when help (in terms of providing information) is needed – might impair satisfying social contacts and appreciation. However, the results show no difference between multiple jobbers and employees with one job regarding the effect of paucity of information on satisfaction with rather structural aspects of the task, with manifest aspects referring to material incentives and with developmental experiences that facilitate the growth. Recall that individuals may have various motives for working at several jobs, including economic needs or desires to broaden job opportunities. Motives might then affect reactions to the paucity of information and aspects of job satisfaction. Therefore, future studies should include the heterogeneous nature of multiple jobbers in the analyses.

Interaction effects did not depend on how much time employees spent at work (or more specific: in their main occupational activity) and in the organization of the main occupational activity. Previous research on part-time work suggests that spending less time in a work place might lead to being less included in the team and getting less challenging tasks (e.g., Rauchert et al., 2016). However, our additional analyses showed that effects on employees’ satisfaction in general and with the social climate could not be reduced to spending less time at the main occupational activity. Working hours did not enhance the effect of the paucity of information on job satisfaction in general or with the social context.

### Strengths and Limitations

This is the first study examining the paucity of information within the scope of holding multiple jobs. The data are based on a representative sample of German employees. Although effects are small, the paucity of information was found to be practically significant with respect to job satisfaction over and above workload and autonomy as important working conditions. However, there are some limitations to our study that should be acknowledged. First, results of cross-sectional studies do allow many alternative explanations of the observed effects, as reverse causation cannot be precluded (Zapf et al., 1996). Consequently, future research should replicate effects using a longitudinal design. Second, all measures were based on self-reports of participants, raising the risk of overestimating results due to common method biases (Podsakoff et al., 2003; but see also Semmer et al., 1996; Spector, 2006). Related to this shortcoming, the influence of trait negative affectivity on job stressors and work attitudes has been discussed in the literature several times (Semmer et al., 1996; Spector, 2006); however, it could not explain differences in well-being between different jobs (Schaubroeck et al., 1998). Third, we studied variables only in relation to the self-prescribed main job, which should be most relevant to perceived stress and job satisfaction (Zickar et al., 2004). However, multiple job holding provides an alternative source of valuable work related outcomes. For example, the primary and secondary jobs could have combined work characteristics that affect job satisfaction in the primary job (Betts, 2002). Future studies should account for aspects of all jobs of the incumbents. Moreover, we used a single item to assess the paucity of information. Although single item measures are known to be valid measures (Wanous et al., 1997; DeSalvo et al., 2006), based on the conceptualization of organizational constraints and role ambiguity, task-related information might be a rather broad concept (e.g., including know-how and feedback) that needs to be explored in more detail. Future research should also be aware that too much information might confound task focus and impair well-being.

### Practical Implications

This study adds to the limited research on multiple jobbers and the paucity of information. Our results indicate that the perception of a paucity of information is negatively related to job satisfaction, especially for multiple as compared to single jobbers. Job satisfaction is known to be associated with performance, work behavior and mental health (Faragher et al., 2005; Fried et al., 2008). Thus, dissatisfaction might lead to severe consequences for both person and organization. Taking into account the rise of new (communication) technologies (e.g., Kompier, 2006) the paucity of information seems to be an important, however, widely neglected topic in modern society. The findings have practical implications: They underline the importance of providing adequate information to employees in general, not only performance feedback but also all information needed to accomplish tasks. Employees expect their supervisors and colleagues to provide them with necessary information (Tschan et al., 2009). Multiple jobbers who lack information are especially likely to be less satisfied with the workplace social climate. In general, organizations should create a communication climate that is characterized by trust, transparency and openness. A transparent information system integrated into this climate could help to find the right source of information in case the person is aware that some information is missing. Supervisors as well as colleagues should also be sensitive to provide all necessary information and to double-check that the given information could be remembered. Effective leadership particularly requires skillful communication. Hence, especially supervisors should be trained to be good communicators. In particular, quantitative information places such a high load on working memory that it requires repeated updating to ensure accuracy; thus strategies such as providing explicitly encoded information might enhance the accuracy of information transmission. Strategies like providing information explicitly encoded as to be transmitted – for instance, “John, please note the third update on XY, as constituted on 1 January 2017” instead of “Once again, we talked about an update on XY today” – might increase the accuracy of the information transmission process (Morris et al., 1977; Bogenstätter et al., 2009). A clearer information transmission process could enable employees to better coordinate their duties. In sum, employees, especially multiple jobbers, will have more job satisfaction if they receive all necessary job-related information.

## Ethics Statement

The study was performed according to requirements: participants were informed of their rights and guaranteed anonymity. All participants gave informed consent in accordance with the Declaration of Helsinki.

## Author Contributions

LH and MK developed the concept. KO, BF, and MK structured the ideas and MK did the analyses and wrote the first draft. All authors read and approved the final manuscript.

## Conflict of Interest Statement

The authors declare that the research was conducted in the absence of any commercial or financial relationships that could be construed as a potential conflict of interest.
